# *Trichinella* Infection in Wildlife of Northeast of Iran

**Published:** 2012

**Authors:** H Borji, H Sadeghi, GhR Razmi, E Pozio, G La Rosa

**Affiliations:** 1 Department of Pathobiology, School of Veterinary Medicine, Ferdowsi University of Mashhad, Mashhad, Iran; 2 Department of Infectious, Parasitic and Immunomediated Diseases, Istituto Superiore di Sanita‘, viale Regina Elena, Rome, Italy

**Keywords:** *Trichinella britovi*, Epidemiology, Carnivores, Iran

## Abstract

**Background:**

The objective of this investigation was to detect the presence of *Trichinella* in some carnivores of Mashhad in northeast of Iran and to identify *Trichinella* species circulating in this area.

**Methods:**

The present study was carried out using muscle tissue collected from 120 stray dogs, 26 wild boars, 25 rodents, two foxes and two hyenas captured in Mashhad City, province of Khorasan Razavi, Iran.

**Results:**

*Trichinella* larvae were detected in three stray dogs by artificial digestion and compression. All larvae were identified as *T. britovi* using multiplex PCR.

**Conclusion:**

This is the first report of identification of *T. britovi* in stray dog in Iran.

## Introduction

Trichinellosis is one of the most important foodborne parasitic zoonoses caused by ingestion of undercooked meat harboring parasites of the genus *Trichinella*. Infection by *Trichinella* spp. has been detected in domestic and wild animals throughout the world, with the exception of Antarctica, where there is no record of the parasite (1–3). The genus *Trichinella* consists of 12 species that all of them are genetically and biologically delineated into two distinct clade characterized by the presence or absence of an intramuscular collagen capsule (4). Human trichinellosis outbreaks occur in many parts of the world, and it has been estimated that as many as 11 million people are infected with this parasite (5). Global distribution of *Trichinella* in conjunction with varying cultural eating habits, represent the main factor favoring human infections.

In Iran, human cases are rare and there are only two published reports of human infections (6). This is mainly due to Muslim beliefs based on not consuming pork and the meat of some other animal species. Furthermore, *Trichinella* was first detected in wild boar and later in seven carnivorous mammalian species and a rodent (7). This parasite has been detected in carnivorous mammals of the Caspian region, Isfahan, Ardabil and Khuzestan in the central and west of Iran (6, 7). However, there is no data available on *Trichinella* infection in the northeast of Iran. Moreover, most of what we know about the prevalence of *Trichinella* in Iran comes from case report studies. It is presently unclear how many carnivores are infected by this parasite and which species of *Trichinella* is prevalent in animals of this area.

The objective of this investigation was to detect the presence of *Trichinella* in some carnivores of Mashhad in northeast of Iran and to identify *Trichinella* species circulating in this area.

## Materials and Methods

### Study area

**Fig. 1:**
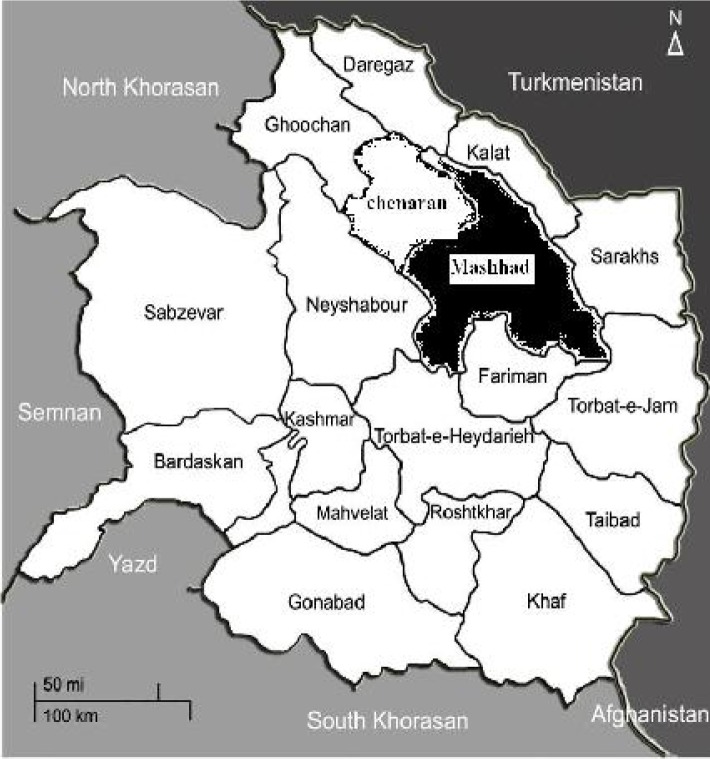
Map of Khorasan Razavi Province showing Mashhad County where the present study was carried out

Mashhad is located in northeast of Iran with a human population of 2.5 million. This city is the second largest holy city in the world which attracts more than 20 million tourists and pilgrims annually. This area is located at 36.20° latitude and 59.35° east longitude, in the valley of the Kashaf River near Turkmenistan, between the two mountain ranges of Binalood and Hezar-masjed (Fig. 1). Annual precipitation is about 250 mm per year. Mashhad also has wetter and drier periods with the bulk of the annual precipitation falling between the months of December to May.

### Animals and Sampling

Totally 120 stray dogs, 26 wild boars, 25 rodents, two foxes and two hyenas were captured in Mashhad, Khorasan Razavi Province, northeast of Iran during October 2010 to June 2011 with permission from the Iranian Environmental Health Organization. From each animal, more than 10 g of muscular tissue were trimmed of tongue; diaphragm and forearm. The samples were preserved in ice during transportation to the laboratory and examined after sampling as soon as possible.

### Direct detection of larvae

Our laboratory research was based on standard artificial digestion techniques according to the standard protocol(8) and compression.

After digestion of muscle tissues, the sediment was allowed to settle and was washed several times. The sediment from the last washing was examined for larvae under a dissection microscope. When the sediment contained *Trichinella* larvae, the procedure was repeated using the individual samples.

### Experimental infection of rat

As a confirmatory study, two rats were orally inoculated by administering 1 ml of 0.9% saline solution containing 300 *Trichinella* larvae by gavage. *Trichinella* larvae were detected in the muscular tissues after 45 days post infection using compression between 2 slides and artificial digestion.

### Histopathological examination

Portions of the tongue, diaphragm and forearm muscles of positive samples were fixed in 10% phosphate-buffered formalin, embedded in low-fusion paraffin, cut in 4-mm-thick sections and finally stained with hematoxylin–eosin and examined under a light microscope.

### Molecular identification of Trichinella spp

*Trichinella* spp. larvae recovered by artificial tissue digestion from positive stray dogs were washed in saline, preserved in 90% ethyl alcohol, and submitted to the International *Trichinella* Reference Center (ITRC, www.iss.it/site/*Trichinella*) in Rome, Italy for genotyping. Individual *Trichinella* spp. larvae were identified by multiplex PCR analysis following the protocol described by Pozio and La Rosa (9). Briefly, DNA was extracted from ten individual worms of each samples; PCR was performed using ExTaq DNA polymerase (Takara) in 50 ml containing 1.5 mM MgCl2, 200 mM dNTPs, and 50 pmol of primer pairs I, II, III, IV and V (Table 1) and 0.5 unit of ExTaq DNA polymerase. The PCR-amplified fragments from purified DNA were visualized by agarose gel electrophoresis (2.0% standard agarose). Single *Trichinella* spp. larvae from one reference strain for each taxa, were used for comparison: *T. spiralis*, *T. nativa*, *T. pseudospiralis*, *T. britovi* and *T. nelsoni*.

**Table 1:** Details of Multiplex PCR primer implemented in the satudy

Primer pairs	Location in DNA	Sequence
I	ESV	5’-GTT.CCA.TGT.GAA.CAG.CAG.T-3’
		5’-CGA.AAA.CAT.ACG.ACA.ACT.GC-3’
II	ITS1	5’-GCT.ACA.TCC.TTT.TGA.TCT.GTT-3’
		5’-AGA.CAC.AAT.ATC.AAC.CAC.AGT.ACA-3’
III	ITS1	5’-GCG.GAA.GGA.TCA.TTA.TCG.TGT.A-3’
		5’-TGG.ATT.ACA.AAG.AAA.ACC.ATC.ACT-3’
IV	ITS2	5’-GTG.AGC.GTA.ATA.AAG.GTG.CAG-3’
		5’-TTC.ATC.ACA.CAT.CTT.CCA.CTA-3’
V	ITS2	5’-CAA.TTG.AAA.ACC.GCT.TAG.CGT.GTT.T-3’
		5’-TGA.TCT.GAG.GTC.GAC.ATT.TCC-3’

## Results

**Fig. 2:**
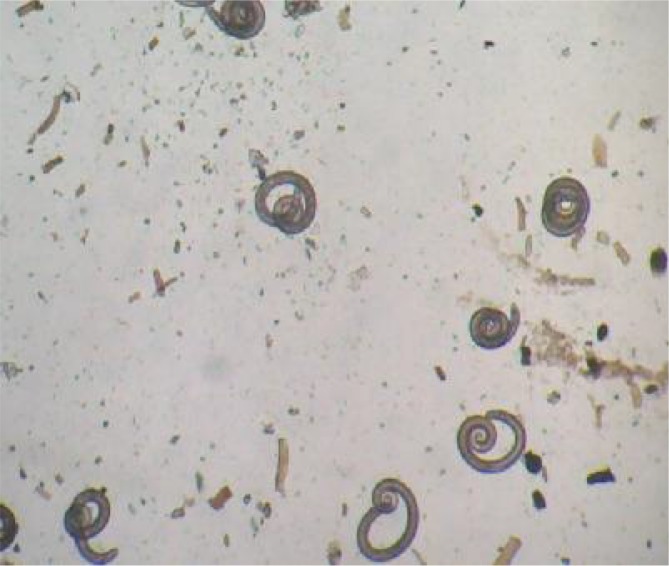
Encapsulated *Trichinella* spp. after digestion of muscle (Original)

**Fig. 3:**
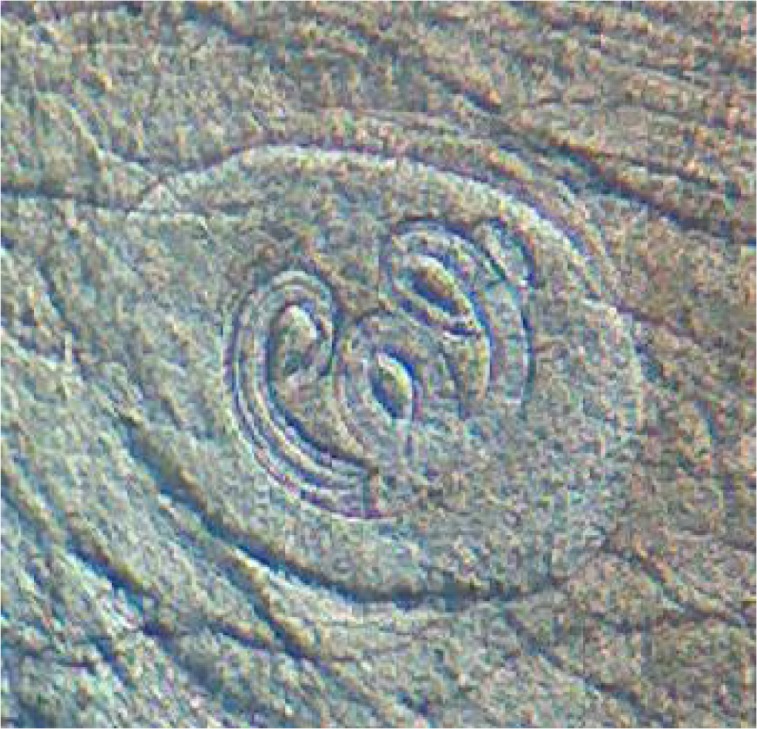
Encapsulated *Trichinella* spp. after compressing (Original)

In none of the 26 wild boars, 25 rodents, two foxes and two hyenas *Trichinella* larvae were found using artificial digestion and compression but, *Trichinella* larvae were found in three of 120 stray dogs (Fig. 2, 3). Ten larvae were recovered after pooled sample digestion. *Trichinella* larvae were visible inside thick collagen capsules by direct microscopic examination of muscle tissue (Fig. 3). Moreover, *Trichinella* larvae were detected after 45 days post experimental infection in two rats using compression and artificial digestion.

**Fig. 4:**
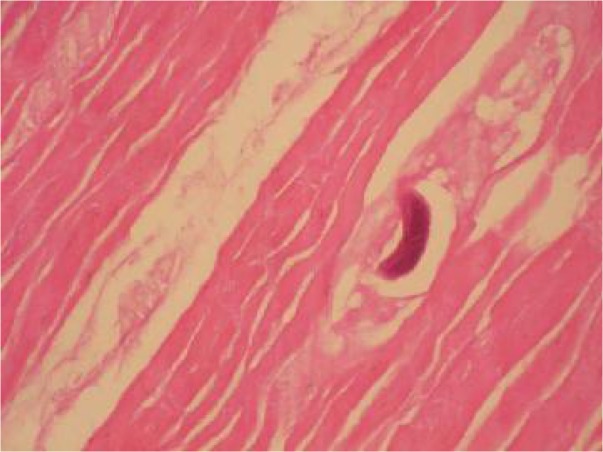
Histopathology section of skeletal muscle that shows encapsulated *Trichinella* spp. H&E (160×) (Original)

In the histopathological examination of the muscles of three stray dogs, encapsulated *Trichinella* larvae were observed (Fig. 4).

**Fig. 5:**
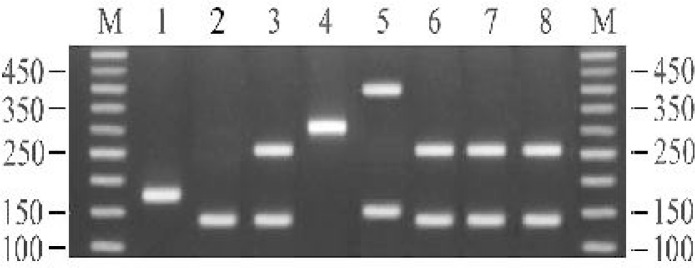
Agarose gel separation of multiplex PCR products from: (1) *T. spiralis* (2) *T. native* (3) *T. britovi* (4) *T. pseudospiralis* (5) *T.nelsoni* (6-8) *T. britovi* from stray dogs of Mashhad, Iran

Banding patterns from multiplex PCR amplifications of the *Trichinella* isolates showed that all of positive stay dogs were infected with *T. britovi* (Fig. 5).

## Discussion

Our results demonstrated for the first time the infection of stray dogs with *T. britovi* in Iran. Presence of *T. britovi* was formerly reported in two previous studies (7, 9) i.e. a leopard (*Panthera pardus saxicolor*) and also in a wild boar in north and west of Iran, respectively. In only one previous study (10), based on morphological characteristics, *T. spiralis* is reported in Iran.

According to religious believes, consumption of pork and wild boar meat in Iran is forbidden, therefore, epidemiology of *Trichinella* has rarely been investigated. These results indicate that *Trichinella* spp. infection has been documented in jackals, red foxes, stray dogs, brown bears, wild cats, wild boars, stripped hyena and in one rodent (*Meriones persicus*) of the north and central region of Iran (11, 12). Recently, *T. murrelli* has been reported in a wild boar (13), but further work indicated that incorrect sequencing of *Trichinella* has lead to misidentification and it was *T. britovi* (9). It should be noted that *T. murrelli* circulates in North America and it has only been detected once outside of this continent, in particular, in a horse slaughtered in France which was imported from USA (9). No information is available on *Trichinella* spp. infection in domestic animals of Iran. Moreover, a doubtful case of human infection was reported based on clinical symptoms, history of eating undercooked wild boar meat, as well as presence of low titer circulating antibody in the serum of the patient (14). Recently, by using molecular methods, a case of trichinellosis in a family was confirmed in Tehran with a history of consumption wild boar meat infected with *T. britovi* (6). In addition, human infections caused by *T. britovi* due to consumption of free-ranging pigs, game and horse meat, have been documented in France, Italy, Spain and Turkey (15, 16). Since females of *T. britovi* produce fewer number of newborn larvae than *T. spiralis*, the clinical course is benign and death has not been documented (1). In general, our results reflects what we believe to be *T. britovi* the most prevalent species in Iran, but *T. spiralis*, if still present, is probably restricted to a small area. Indeed, *T. britovi* is highly adapted to carnivores and *T. spiralis* to swine (17). It is better to know that the Russian authors named as *T. nelsoni* all the *Trichinella* taxa which today are identified as *T. britovi*, *T. nelsoni* and *Trichinella T8*. Isolates of *T. nelsoni* are present only in eastern and southern Africa (from Kenya to South Africa).

## Conclusion

Due to social, economic and religious factors in Iran, the risk of trichinellosis infection among humans is low but sporadic cases still exists among people that consume wild boar meat in some areas.
